# Improving Influenza Vaccination Rate among Primary Healthcare Workers in Qatar

**DOI:** 10.3390/vaccines5040036

**Published:** 2017-10-10

**Authors:** Khalid H. Elawad, Elmoubasher A. Farag, Dina A. Abuelgasim, Maria K. Smatti, Hamad E. Al-Romaihi, Mohammed Al Thani, Hanan Al Mujalli, Zienab Shehata, Merin Alex, Asmaa A. Al Thani, Hadi M. Yassine

**Affiliations:** 1 Primary Health Care Corporation, Doha 26555, Qatar; kelawad@phcc.gov.qa (K.H.E.); mvalisto@phcc.gov.qa (D.A.A.); halmujalli@phcc.gov.qa (H.A.M.); zshehata@phcc.gov.qa (Z.S.); malex@phcc.gov.qa (M.A.); 2 Ministry of public Health, Doha 42, Qatar; eabdfarag@moph.gov.qa (E.A.F.); halromaihi@moph.gov.qa (H.E.A.); malthani@MOPH.GOV.QA (M.A.T.); 3 Biomedical Research Center, Qatar University, Doha 2713, Qatar; msmatti@qu.edu.qa (M.K.S.); aaja@qu.edu.qa (A.A.A.T.)

**Keywords:** influenza, vaccine, health care workers

## Abstract

The purpose of this study was to improve influenza vaccination, and determine factors influencing vaccine declination among health care workers (HCW) in Qatar. We launched an influenza vaccination campaign to vaccinate around 4700 HCW in 22 Primary Health Care Corporation (PHCC) centers in Qatar between 1st and 15th of November, 2015. Our target was to vaccinate 60% of all HCW. Vaccine was offered free of charge at all centers, and information about the campaign and the importance of influenza vaccination was provided to employees through direct communication, emails, and social media networks. Staff were reported as vaccinated or non-vaccinated using a declination form that included their occupation, place of work and reasons for declining the vaccine. Survey responses were summarized as proportional outcomes. We exceeded our goal, and vaccinated 77% of the target population. Only 9% declined to take the vaccine, and the remaining 14% were either on leave or had already been vaccinated. Vaccine uptake was highest among aides (98.1%), followed by technicians (95.2%), and was lowest amongst pharmacists (73.2%), preceded by physicians (84%). Of those that declined the vaccine, 34% provided no reason, 18% declined it due to behavioral issues, and 21% declined it due to medical reasons. Uptake of influenza vaccine significantly increased during the 2015 immunization campaign. This is attributed to good planning, preparation, a high level of communication, and providing awareness and training to HCW with proper supervision and monitoring.

## 1. Introduction

Influenza is a major health concern, and it results in a significant clinical and socioeconomic burden worldwide. The virus can be is easily transmitted, even from those that exhibit little or no clinical symptoms. The risk of serious illness and complications from influenza infection increase for those with underlying health conditions, elderly people, infants and pregnant women [1].

Nosocomial influenza outbreaks occur in almost all types of health care centers worldwide, and their consequences on patients and health care centers in terms of morbidity, mortality and costs are substantial [2]. The source of infection in such outbreaks is often unknown, since the visitors, patients, or even the health care workers (HCW) themselves could transmit the virus to susceptible individuals within the health care center [3].

Despite the strong recommendations from the WHO and CDC, and the fact that influenza vaccination of HCW improves patient and employee safety, influenza vaccine uptake among HCW remains low, worldwide [4]. Availability of influenza vaccines at no cost and at work sites, education aimed at increasing awareness about the importance of annual influenza vaccinations and, most importantly, switching to a mandatory influenza vaccination policy, could lead to a high and sustained vaccine coverage among HCW.

In the year 2014, the influenza vaccination rate among HCW in Primary Health Care Corporation (PHCC) centers in Qatar was very low (35%; PHCC records). Accordingly, health care officials at the Ministry of Public Health (MoPH) decided to launch an influenza vaccination campaign in 2015 to vaccinate at least 60% of all PHCC employees in Qatar. Influenza season is very unpredictable, and varies from year to year. In Qatar, influenza activity can begin as early as of September and continues to occur until May. Accordingly, the campaign was launched on the 1st of November 2015, and lasted for two weeks. The campaign was very successful, and significantly increased influenza vaccine coverage among HCW in Qatar compared to previous years.

## 2. Materials and Methods

The PHCC is a non-profitable government entity that runs under the MoPH-Qatar, and is focused on providing primary and essential health care services. Currently, the PHCC is operating through 22 primary health care centers distributed into three regions: Central, Western, and Northern. The corporation employs around 4700 staff, including physicians, pharmacists, nurses, technicians, aids and administrators. Staff were defined as those who were permanently or temporarily employed, and who had worked at least one shift at their specific center. All staff were targeted for influenza vaccination through a progressive campaign that lasted 15 days between 1 and 15 November, 2015. The campaign used simple messages, “Save lives—Immunize”, “Healthcare workers need a flu vaccine too” and “The flu ends with you”. The target was to vaccinate at least 60% of employees. Participation was voluntary, and anonymity of respondents was preserved. Approval to perform the study was obtained from the MoPH. Information about the campaign and the importance of influenza vaccination was provided through: (a) Employee communications networks (e.g., staff meetings); (b) emails (all HCW); (c) SMS (for all employees), as well as flyers and brochures. The PHCC intranet, Twitter, and Facebook were also used to provide health education messages and educational materials. Vaccine was offered freely at all centers, roving carts, drive through clinics, after hour clinics and at regular meetings. Flyers about the influenza virus, influenza vaccine types, components, safety and risks were made available to employees. Participants received one intramuscular shot (0.5 mL) of 2015–2016 Quadrivalent Vaccine-Fluarix™ Tetr (GlaxoSmithKline Biologicals; Dresden, Germany) or 2015–2016 trivalent influenza vaccine-INFLUVAC (Abbott Biologicals, Olst, The Netherlands) for pregnant women. Staff were reported as either vaccinated or non-vaccinated using a declination form that included their occupation, place of work and reasons for declining the vaccine. Demographic data about gender and age were not collected, and thus were not included in the analysis. Survey responses were summarized as proportional outcomes. Statistical analysis was performed using GraphPad Prism 7 software (GraphPad Software, Inc., La Jolla, CA, USA). Odds ratios with a 95% confidence interval were calculated to measure associations between factors of interest. Results with *p*-value < 0.05 were considered statistically significant.

## 3. Results

**Figure 1:**
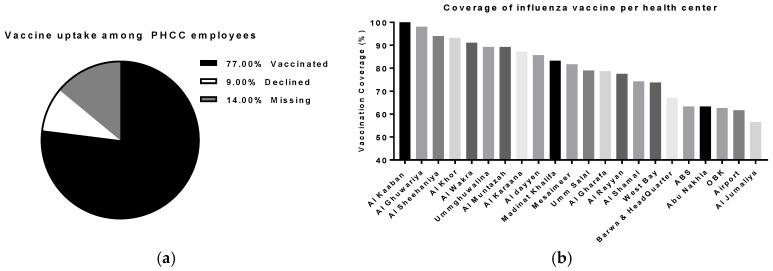
Influenza vaccine coverage in primary health care centers in Qatar, 2015. (**a**) Total coverage of influenza vaccine among PHCC employees; (**b**) Coverage of influenza vaccine among PHCC employees per health center.

The campaign approached all staff members (*n* = ~4700), of which 4082 (86% of total staff) participated in the study, including 1291 administrators, 1061 nurses, 546 aides, 540 physicians, 401 technicians, and 243 pharmacists. The proportion of staff vaccinated in our health centers following this campaign was much higher (77%; *n* = 3629) than we had previously achieved (<50%, PHCC records), and significantly higher than a previously reported study that estimated the uptake of pandemic H1N1 in 2011 [5]. Only nine percent (*n* = 453) declined the vaccine, and the remaining 14% were either on leave or had already been vaccinated (Figure 1a).

On average, the coverage of influenza vaccination per health center was around 79.5%. The highest vaccination rate was recorded in Al Kaaban center-North (100%; *n* = 36), while the lowest rate was recorded in Al Jumaliya center-West (56.5%; *n* = 13). The percentage of vaccinated staff in the headquarters was 67.1%; *n* = 557 (Figure 1b; Table 1).

**Figure 2:**
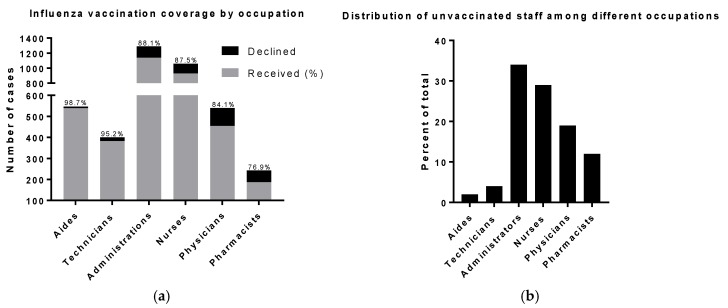
Influenza vaccination by occupation in PHCC. (**a**) Percent of PHCC respondents vaccinated by job category; (**b**) Distribution of unvaccinated staff among different occupations.

The health care aide profession was significantly related to high vaccine uptake. Aides were the most likely to get vaccinated, about 11.1 times higher than other professions (98.7% (*n* = 539); 95% CI 5.4–23.4, *p* < 0.0001), followed by technicians (95.2% (*n* = 382); OR: 2.7; 95% CI 1.7–4.3; *p* < 0.0001). Pharmacists and physicians were the least likely to take the vaccine (73.2%; (*n* = 187); OR: 0.4; 95% CI 0.3–0.5, *p* = 0.0002), (84% (*n* = 454); OR: 0.6; 95% CI 0.5–0.8; *p* < 0.0001), respectively. There were no significant differences between administrators and nurses with regard to vaccine uptake (Figure2a; Table 2). Among those who declined to take the vaccine, administrators had the highest rate (34%; *n* = 153), followed by nurses (29%; *n* = 132), and the lowest rate was for aides (2%; *n* = 7) (Figure 2b).

While 35% (*n* = 157) of those who declined to take the vaccine provided no reason for their decision, 18% (*n* = 83) declined the vaccine due to behavioral issues, including fear of needles, the myth of getting avian flu, or the belief of their having strong natural immunity. Other reasons for declining the vaccine included sickness (5.5%; *n* = 25), pregnancy (7.0%; *n* = 32), allergy to eggs (6.2%; *n* = 28), being on treatment (1.1%; *n* = 5) and breast-feeding (1.3%; *n* = 6). Among the decliners, technicians and nurses reported the highest rate of vaccination in the previous year. We found no correlation between profession and reasons of vaccine non-compliance (Table 3).

## 4. Discussion

A PHCC center receives, on average, 250,000 patients every month. Such a high number of patients increases the risk of nosocomial infections, and frontline HCW have a duty of care to protect their patients and service users from such infections. Although influenza vaccine uptake has been low for the past years, the 2015 campaign significantly delivered the vaccine to 77% of HCW in the PHCC centers. This significant increase in influenza vaccination could be attributed to many factors: (1) Extended vaccination campaign—15 days in 2015, compared to only 3 days in 2014; (2) Enhanced communication with employees through direct contact, emails, text messages, brochures and flyers, as well as social media; (3) Employment of peer vaccinators to help stimulate interest in vaccination; (4) Addressing perceived barriers about influenza vaccine safety—the low risk of serious complication from influenza vaccination and the risk of severe complications from natural infection; and (5) Providing the vaccine free-of-charge at all centers.

The outcomes of this study indicate that we developed and executed an effective strategy for improving influenza vaccine uptake in primary health care facilities that depends mainly on increasing vaccine availability free of charge, and implementing a social marketing campaign. Nonetheless, our improved rate of vaccination for HCW did not reach the rate recorded in a recent non-mandatory program implemented in Japan [6]. Their strategy led to 97% vaccination uptake among HCW, but it was implemented at a smaller scale (single-site hospital), and involved interviewing non-compliant staff by the hospital executive. Our program spanned a larger number of employees (4700) at multiple centers (*n* = 22), and did not include direct communication between employees and their higher executives. Nonetheless, vaccination rate among HCW in Qatar was comparable to previously reported rates in United States (US) between 2013–2014 (75.2%) and 2014–2015 (77.3%). Similar rates of influenza vaccination among HCW were also reported in non-mandatory programs implemented in Australia in 2014 [7]. However, lower rates have been reported in other Arabic Gulf countries, including UAE (24.7%), KSA (38%), Oman (46.4%) and Kuwait (67.2%) [8,9]. Interestingly, the highest vaccination rate in US health care centers was reported amongst pharmacists (95.3%), and the lowest was reported amongst aids (64.4%), which is totally the opposite of what we have reported in this study [10,11].

Reasons for staff non-compliance with our dedicated program were partially assessed in the current study and shall be considered in planning for future programs. Interestingly, physicians were less likely to take the vaccine (84%) compared to other professions, such as aides (98.1%) and technicians (95.2%). Similar observations were reported among pediatric HCW in Qatar in 2015 [12]. In that study, which assessed the attitude and perceptions toward influenza vaccination among 230 pediatric HCW at the main tertiary teaching hospital in Qatar, allied health care practitioners were more likely to get the vaccine (69%) than physicians (66%). It is unclear why physicians are less likely to receive the vaccine compared to other occupations, and this shall be assessed in future campaigns.

Ideally, it is hoped that influenza vaccination among HCW will reach 100%; nevertheless, several studies have suggested that a vaccination rate of 80% among HCW would provide herd immunity, and significantly reduce influenza transmission within health care facilities [13]. Accordingly, the US Department of Health and Human Services has set a goal of reaching a 90% vaccination rate by 2020 [14]. Mandatory immunization programs might be necessary to increase vaccination rate among PHCC HCW, especially for those that work in PHCC with people at high risk of developing influenza-related complications (children younger than 5 years, adults 65 years of age or older, pregnant women, and others), or people who have medical condition such as asthma, COPD, sickle cell anemia, and diabetes. This would mostly likely require an understanding of cultural attitudes towards vaccines and their effectiveness. A future campaign will target more HCW from different medical disciplines, in addition to providing multidisciplinary educational approaches to address perceived barriers about influenza vaccine.

## 5. Conclusions

In summary, our target was to vaccinate 60% of all HCW in Primary Health Care Centers in Qatar. We exceeded our goal, and vaccinated 77% of the targeted population. This success could be attributed to good planning, a high level of communication with all employees and, most importantly, providing the vaccine free-of-change for a prolonged period, compared to previous years. Reasons for staff non-compliance with our dedicated program were partially assessed in the current study, and shall be considered in planning for future programs. Finally, HCW act as role models that influence public attitudes and behaviors with regard to health issues. Considering the increase in influenza vaccinations among health care practitioners in Qatar, we hope that this will positively influence the public to consider annual influenza vaccination.

## Figures and Tables

**Table 1:** Coverage of influenza vaccination per primary healthcare center (*n* = 22) in Qatar.

PHCC Employees Location	Percent Given (No.)
Airport	61.6 (153)
Ummghuwalina	89.3 (193)
Al Wakra	91.1 (237)
Al Muntazah	89.2 (224)
West Bay	73.8 (178)
Al Ghuwariya	98.1 (52)
Al Shamal	74.2 (95)
Al Khor	93.3 (141)
Al Rayyan	77.6 (118)
Al Karaana	87.2 (41)
Al Sheehaniya	94 (175)
Abu Nakhla	63.3 (128)
OBK	62.6 (168)
Al dayyen	85.7 (120)
Umm Salal	79 (151)
Al Gharafa	78.7 (211)
Al Kaaban	100 (36)
Madinat Khalifa	83.3 (221)
Al Jumaliya	56.5 (13)
Barwa & Head Quarter	67.1 (557)
ABS	63.3 (180)
Mesaimeer	81.7 (237)
**Average (Total)**	**79.5 (3629)**

**Table 2:** Vaccination compliance in association with participants’ occupation.

Occupation	Percent Given (No.)	Odds Ratio ^a^	95% CI ^b^	*p* Value
Aide	98.7 (539)	11.1	5.4–23.4	<0.0001
Administration	88.1 (1138)	0.9	0.73–1.1	ns
Technician	95.2 (382)	2.7	1.7–4.3	<0.0001
Nurse	87.6 (929)	0.8	0.3–1.0	ns
Physician	84.1 (454)	0.6	0.5–0.8	0.0002
Pharmacist	76.9 (187)	0.4	0.3–0.5	<0.0001
**Average (Total)**	**88.9 (3629)**			

^a^ Calculated for each occupation versus others; ^b^ CI, confidence interval.

**Table 3:** Main reasons reported for not receiving influenza vaccinations by occupation.

Reason	Occupation					
	Aide	Admin	Technician	Nurse	Physician	Pharmacist
No reason provided	71.4 (5)	54.3 (83)	5.3 (1)	24.2 (32)	32.6 (28)	14.3 (8)
To avoid side effects	14.3 (1)	6.5 (10)	5.3 (1)	9.8 (13)	16.3 (14)	12.5 (7)
Vaccinated previous year	0 (0)	7.8 (12)	26.3 (5)	28.8 (38)	15.1 (13)	5.4 (3)
Behavior	0 (0)	14.4 (22)	36.8 (7)	7.6 (10)	19.8 (17)	48.2 (27)
Sick	0 (0)	3.3 (5)	10.5 (2)	9.1 (12)	4.7 (4)	3.6 (2)
Pregnant	0 (0)	6.5 (10)	0 (0)	10.6 (14)	4.7 (4)	7.1 (4)
Egg allergy	14.3 (1)	5.2 (8)	15.8 (3)	4.5 (6)	5.8 (5)	8.9 (5)
On treatment	0 (0)	1.3 (2)	0 (0)	1.5 (2)	1.2 (1)	0 (0)
Breast feeding	0 (0)	0.7 (1)	0 (0)	3.8 (5)	0 (0)	0 (0)
**Total Percent (N)**	**100 (7)**	**100 (153)**	**100 (19)**	**100 (132)**	**100 (86)**	**100 (56)**
